# Dysregulated microRNA Clusters in Response to Retinoic Acid and CYP26B1 Inhibitor Induced Testicular Function in Dogs

**DOI:** 10.1371/journal.pone.0099433

**Published:** 2014-06-09

**Authors:** Vanmathy R. Kasimanickam, Ramanathan K. Kasimanickam, William S. Dernell

**Affiliations:** Department of Veterinary Clinical Sciences, Washington State University, Pullman, Washington, United States of America; Ecole Normale Superieure de Lyon, France

## Abstract

Spermatogenesis is a multistep synchronized process. Diploid spermatogonia differentiate into haploid spermatozoa following mitosis, meiosis and spermiogenesis. Division and differentiation of male germ cells is achieved through the sequential expression of several genes. Numerous mRNAs in the differentiating germ cells undergo post-transcriptional and translational regulation. MiRNAs are powerful negative regulators of mRNA transcription, stability, and translation and recognize their mRNA targets through base-pairing. Retinoic acid (RA) signaling is essential for spermatogenesis and testicular function. Testicular RA level is critical for RA signal transduction. This study investigated the miRNAs modulation in an RA- induced testicular environment following the administration of all-trans RA (2 µM) and CYP26B1- inhibitor (1 µM) compared to control. Eighty four canine mature miRNAs were analyzed and their expression signatures were distinguished using real-time PCR based array technology. Of the miRNAs analyzed, miRNA families such as miR-200 (cfa-miR-200a, cfa-miR-200b and cfa-miR-200c), Mirlet-7 (cfa-let-7a, cfa-let-7b, cfa-let-7c, cfa-let-7g and cfa-let-7f), miR-125 (cfa-miR-125a and cfa-miR-125b), miR-146 (cfa-miR-146a and cfa-miR-146b), miR-34 (cfa-miR-34a, cfa-miR-34b and cfa-miR-34c), miR-23 (cfa-miR-23a and cfa-miR-23b), cfa-miR-184, cfa-miR-214 and cfa-miR-141 were significantly up-regulated with testicular RA intervention via administration of CYP26B1 inhibitor and all-trans-RA and species of miRNA such as cfa-miR-19a, cfa-miR-29b, cfa-miR-29c, cfa-miR-101 and cfa-miR-137 were significantly down-regulated. This study explored information regarding chromosome distribution, human orthologous sequences and the interaction of target genes of miRNA families significantly distinguished in this study using prediction algorithms. This study importantly identified dysregulated miRNA species resulting from RA-induced spermatogenesis. The present contribution serves as a useful resource for further elucidation of the regulatory role of individual miRNA in RA synchronized canine spermatogenesis.

## Introduction

Spermatogenesis is a process of spermatogonial stem cells developing into highly differentiated spermatozoa by proliferation of spermatogonia, meiosis of spermatocytes and spermiogenesis of haploid spermatids. Spermatogenesis is tightly regulated at the transcriptional and post-transcriptional level for the continuous production of spermatozoa. Numerous genes in differentiating germ cells undergo post-transcriptional and translational regulation. However, in mammalian spermatogenesis, levels of gene expression do not always correspond to the levels of protein expression since some mRNAs are translationally repressed at some stages of spermatogenesis [1]. Therefore, enhancement and repression of post-transcriptional regulatory mechanisms are essential to the success of spermatogenesis.

MicroRNAs (MiRNAs) mediate translational repression and degradation of target mRNAs in several biological processes. Therefore, miRNAs are proposed to essentially mediate spermatogenesis by regulating post-transcription. Varieties of miRNAs are differentially expressed in various tissues suggesting the role in their biological functions. Vertebrate testis displays distinct miRNA profiles [2], [3]. Conditional loss of miRNA function via mutation of Dicer enzyme in the mouse causes defects in proliferation of pre-meiotic germ cells [4]. These defects in mitosis highlight the potentially imperative, but uncharacterized, role of miRNAs during early spermatogenesis. Several miRNAs including members of the let-7 family, miR-378, and miR-140 are sexually dimorphic in developing mouse embryo and this sexual dimorphic expression pattern strongly suggests its roles in testis differentiation, development and spermatogenesis [5]. Combining experimental and computational tools for deciphering miRNA functions and their target genes in testis is important for elucidating spermatogenesis precisely.

Retinoic acid (RA) is an active product of vitamin A metabolism and a critical signaling molecule of spermatogenesis. RA signaling is important for both the initiation of differentiation and the entry into meiosis in male germ cells. CYP26B1 is the major catabolizing enzyme of RA in testis, regulates testicular RA level and CYP26B1 inhibitor has better control over male meiosis-specific gene expressions in canine testis than direct administration of RA [6]. Mouse testis deficient in RA shows an arrest of A_al_ spermatogonia to A_1_ spermatogonia transition and differentiation of A_al_ to A_1_ spermatogonia is an essential step of spermatogenesis [7]. Many investigations suggest that RA directly induces spermatogonial differentiation via the expression of numerous RA-targeted genes such as STRA8, SCP3 and DMC1 [6]. MiR-146 is highly down-regulated in differentiated spermatogonia compared to undifferentiated spermatogonia, at this RA-dependent step of spermatogonial differentiation [8]. Further, administration of RA to murine spermatogonia up-regulates the Mirlet7 family of miRNAs and down-regulates members of the Mir-17-92 (Mirc1) and Mir-106b-25 (Mirc3) clusters [9]. RA-treated neuroblastoma cells show prominent expression changes of miR-10a and miR-10b including modulation of several other miRNA species and suggests RA-induced miRNAs regulation [10].

In the present study, we investigated modulation of miRNA clusters in response to exogenous administration of all-trans retinoic acid and CYP26B1 inhibitor in canine testis.

## Methods

### Ethics statement

This study was performed in strict accordance with the ethics and use of the animal species models for research. The protocol was approved by the institutional animal care and use committee of the Washington State University (Protocol Number: 04070-001). Informed consent from owners of animals was obtained for using dogs' testes.

### Study Population

Testes were collected from healthy, medium sized, mixed breed dogs undergoing elective castrations through the Community Practice Service at the Washington State University Veterinary Teaching Hospital. The testes of dogs at the age of 7.5±0.5 mo (Animals  = 3; Treatments/animal  = 3) were used for the testis organotypic culture.

### Chemicals

Dulbecco's Phosphate-Buffered Saline (DPBS, Invitrogen life technologies, Green Island, NY, USA), high glucose Dulbecco's Modified Eagle Medium (DMEM, Invitrogen life technologies, Grand Island, NY, USA), Fetal Bovine Serum (FBS, Thermo Scientific, West Palm Beach, FL, USA), Penicillin-Streptomycin (Sigma-Aldrich Corp., St. Louis, MO, USA), Dimethyl sulphoxide (DMSO, Sigma-Aldrich Corp., St. Louis, MO, USA), all trans-RA (Sigma-Aldrich Corp.,), CYP26B1 inhibitor R115866 (Johnson and Johnson Pharmaceutical Research and Development, New Brunswick, NJ, USA), TRIzol (Invitrogen by life technologies), DNase I enzyme (amplification grade, Invitrogen life technologies), canine specific miScript miRNA kit including miScript II RT Kit, miScript SYBR Green PCR Kit and miScript miRNA PCR Array plates (Qiagen, Valencia, CA, USA) were purchased and utilized in this study.

### Testis culture

Following castration, testes were kept in pre-warmed Dulbecco's PBS at 37°C and brought to the laboratory immediately. To continue organ culture, a sterile environment was strictly maintained and sterile procedures were consistently employed. The testes were decapsulated. The vas deferens, epididymis and vascular structures were removed, the tunica albuginea was cut opened, and small pieces of testicular parenchyma were removed for culture. The testis samples were further cut into smaller pieces (approximately 2 mm^3^). An organotypic membrane cell culture insert (EMD Millipore, Billerica, MA, USA) was used to culture testis explants. Briefly, it has a micro-porous membrane filter at the bottom of the cylindrical polystyrene holder and it sits on legs in cell culture dishes. This culture insert along with the cell culture plate more closely approximated an in vivo environment than a cell culture plate. In this study, 60×15 mm cell culture dishes were used to hold 30 mm culture plate inserts. Approximately 5 mL culture medium was used to culture the testicular fragments and the insert was not completely immersed in the culture medium to keep the fragments at the liquid-air interface. Eight testicular fragments of similar size were placed on the membrane per treatment group to maintain the homogeneity of testicular parenchyma and facilitate sampling. Testicular fragments were positioned at the interface.

### Treatment groups

High glucose Dulbecco's modified Eagle medium with 10% fetal bovine serum and penicillin-streptomycin (2.5 mL of 10,000 IU penicillin and 10 mg streptomycin/mL was added to 500 mL of medium) was prepared to culture explants in an environment with 5% CO2 and 100% humidity at 37°C in a cell/tissue culture incubator. The study was designed for three treatment groups, including control and three animal replicates. The culture medium with testis was treated with exogenous all trans-RA at 2 µM final concentration that was found to be an optimum treatment level in a previous study [11] and CYP26B1 inhibitor R115866 at 1 µM final concentration that was established as a desired concentration for a maximum response to an exogenous small molecule's administration in a previous study [6]. The RA and CYP26B1 inhibitor were dissolved in DMSO, and DMSO treatment alone was used as control. The cultures were incubated for 24 h since these treatments-responses were maximum at 24 h in our previous study [6]. After 24 h of incubation, tissues were collected and homogenized in TRIzol for total RNA isolation.

### RNA extraction

To extract total RNA, all fragments of testicular tissue from each treatment group were homogenized in 1 mL TRIzol in a 1.5 mL micro centrifuge tube using a plastic pestle (Fisher Scientific, Pittsburg, PA, USA). Homogenized tissue samples were incubated for 5 min at room temperature to allow separation of nucleoprotein complexes into the TRIzol. Subsequent addition of chloroform and centrifugation, phase separation was carefully carried out to isolate the aqueous phase containing RNA. Consequently, RNA was precipitated by isopropyl alcohol and washed with 75% ethanol. The RNA pellet was re-suspended in RNase-free water at 60°C. The RNA concentration was measured using a NanoDrop 1000 spectrophotometer (Thermo Scientific) and RNA's quality was determined pure. Sample absorbance was measured at 260 and 280 nm. The ratio of the absorbance at 260 and 280 was approximately 2.0 for all samples. Before downstream applications, all RNA samples were treated with DNase I enzyme (Invitrogen life technologies, Green Island, NY, USA) to degrade any contaminated, single and double stranded DNA.

### Reverse Transcription

A micro Script II RT kit (Qiagen, Frederick, MD, USA) was used for reverse transcription. One µg of RNA samples of controls and treatments were used. Micro Script HiSpec buffer (5×) was used to prepare cDNA for mature miRNA profiling, which was performed with miRNome miScript miRNA PCR Arrays. Template RNA, 10X miScript Nucleics Mix, 5× miScript HiSpec Buffer, and RNase-free water were combined into 20 µL of reverse transcription reaction mix and 1 µg of template RNA was added. The reverse transcription reaction mix was incubated at 37°C for 60 minutes, at 95°C for 5 minutes to inactivate miScript Reverse Transcriptase Mix, and then placed on ice. The mix was diluted with 90 µL of RNase free water and stored at -20°C for further profiling of well characterized canine mature miRNAs.

### Canine Mature miRNA Expression Profiling Using Real-Time Polymerase Chain Reaction (PCR)

Mature miRNA profiling was performed using miScript miRNA PCR Arrays in combination with the miScript SYBR Green PCR Kit (Qiagen, Frederick, MD, USA), which contains miScript Universal Reverse Primer and QuantiTect SYBR PCR Master Mix. Reaction mix for canine miRNome miScript miRNA PCR Arrays was prepared with 2X QuantiTect

SYBR Green PCR Master Mix, 10X miScript Universal Reverse Primer, RNase-free water and template DNA. Twenty five µL of the reaction mix was added to the each well of a 96-well plate, miScript miRNA PCR Array Dog miFinder (Qiagen, Frederick, MD, USA). To activate HotStarTaq DNA Polymerase the plate was heated at 95°C for 15 minutes. Forty cycles of three-step cycling were programmed in a StepOne Plus instrument (Applied Biosystems, Inc., Carlsbad, CA, USA). The step included denaturation at 94°C for 15seconds, annealing at 55°C for 30 seconds and the extension at 70°C for 30 seconds. Fluorescence data was collected during the extension step. Specificity and identity were verified by dissociation curve analysis. Baseline and threshold were set automatically for all PCR runs. CT values were exported as an Excel file for further analyses. Since this array employs completely Quantitative Real Time PCR (qPCR), the experiment did not require validation by single qPCR like other microarray data.

### Dog miRNA PCR Array and Analysis

The Canine miScript miRNA PCR Array plate includes primers for 84 well-characterized mature miRNAs and controls (Table S1). The controls are cel-miR-39-3p (H01 & H02), SNORD61 (H03), SNORD68 (H04), SNORD72 (H05), SNORD95 (H06), SNORD96A (H07), RNU6-2 (H08), miRTC (H09 & H10) and PPC (H11 & H12). SNORDs and RNU6-2 serve as internal normalizers. Two reverse transcription controls and two positive controls ensure the efficiency of the array, the reagents, and the instrument.

### Data Handling and Analyses

Raw CT data in Excel Version 1997–2003 (.XLS file format) was uploaded to the web based software, http://pcrdataanalysis.sabiosciences.com/pcr/arrayanalysis.php. Data QC section was reviewed to assess the PCR reproducibility, reverse transcription efficiency, and to detect genomic DNA contamination in amplified samples of both groups. SNORD61, SNORD68,

SNORD72, SNORD95, SNORD96A and RNU6-2 were selected as housekeeping genes and normalized by arithmetic mean. Data was reviewed for distribution of threshold cycle values and the raw data average in each group. Average CT, 2∧(- CT), fold change, P-value, and fold regulation were calculated by the software and the fold change and P-value results were included in all subsequent graphical analyses. Average CT values were converted to linear 2∧(-CT) values, p-values were calculated using Student's T-test.

### Chromosome distribution and miRNAs-genes integration

Primary miRNAs transcribed from different canine chromosomes were analyzed using miRBase database [12]. Integrative analyses of miRNAs and genes network were elucidated using miRBase, miRDB [13], Target Scan [14] and GeneMANIA [15]. Set of input genes were determined with the target score and total context score respectively from miRDB and Target Scan. An associated network was created with the input genes selected and using miRNA interactions in the algorithm, GeneMANIA. Input genes were established on human orthologous miRNAs for canine species.

## Results and Discussion

Our goal was to identify miRNA clusters associated to RA-dependent spermatogenesis in canine testis and to construct a miRNA-mRNA network consisting of miRNAs, their target genes and their transcription factors. Therefore, we modified the testicular RA level by exogenous administration of RA and CYP26B1 inhibitor in this testis culture study to investigate the miRNA clusters associated with the RA signaling. Actual ability of spermatogenic cells to divide and differentiate in an in vitro culture and their functionality in an in-vitro medium are still in question. There are inherent limitations in culturing and maintaining male germ cells ex-vivo. Nonetheless, a testis organotypic model preserves the true architecture, cell viability and pathway activities. Therefore, the ex-vivo testis culture model is considered a promising model for pharmacological intervention studies.

**Figure 1:**
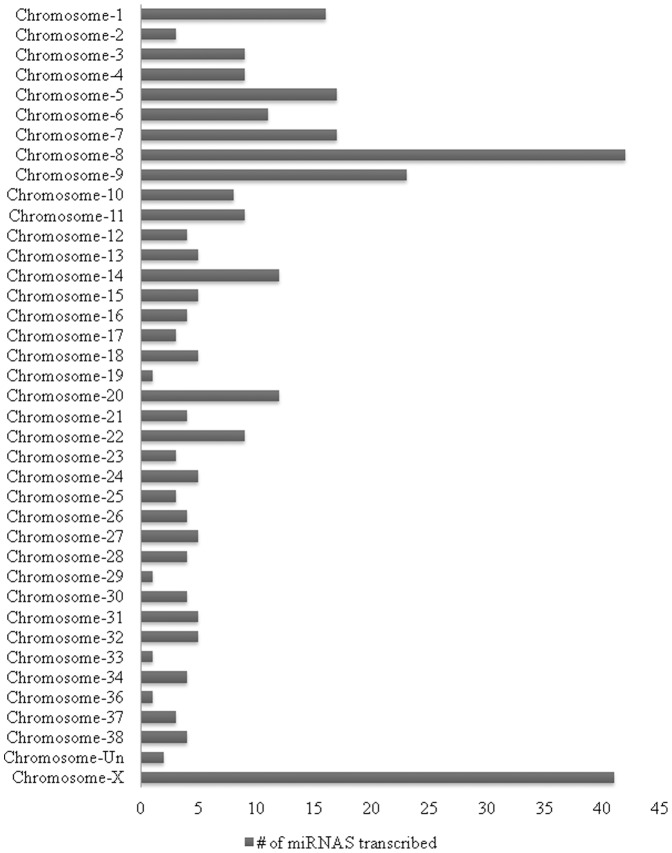
Type of chromosomes from which the number of canine miRNAs (included in the database) are transcribed. Chromosome X and chromosome 8 transcribe several miRNAs.

**Figure 2:**
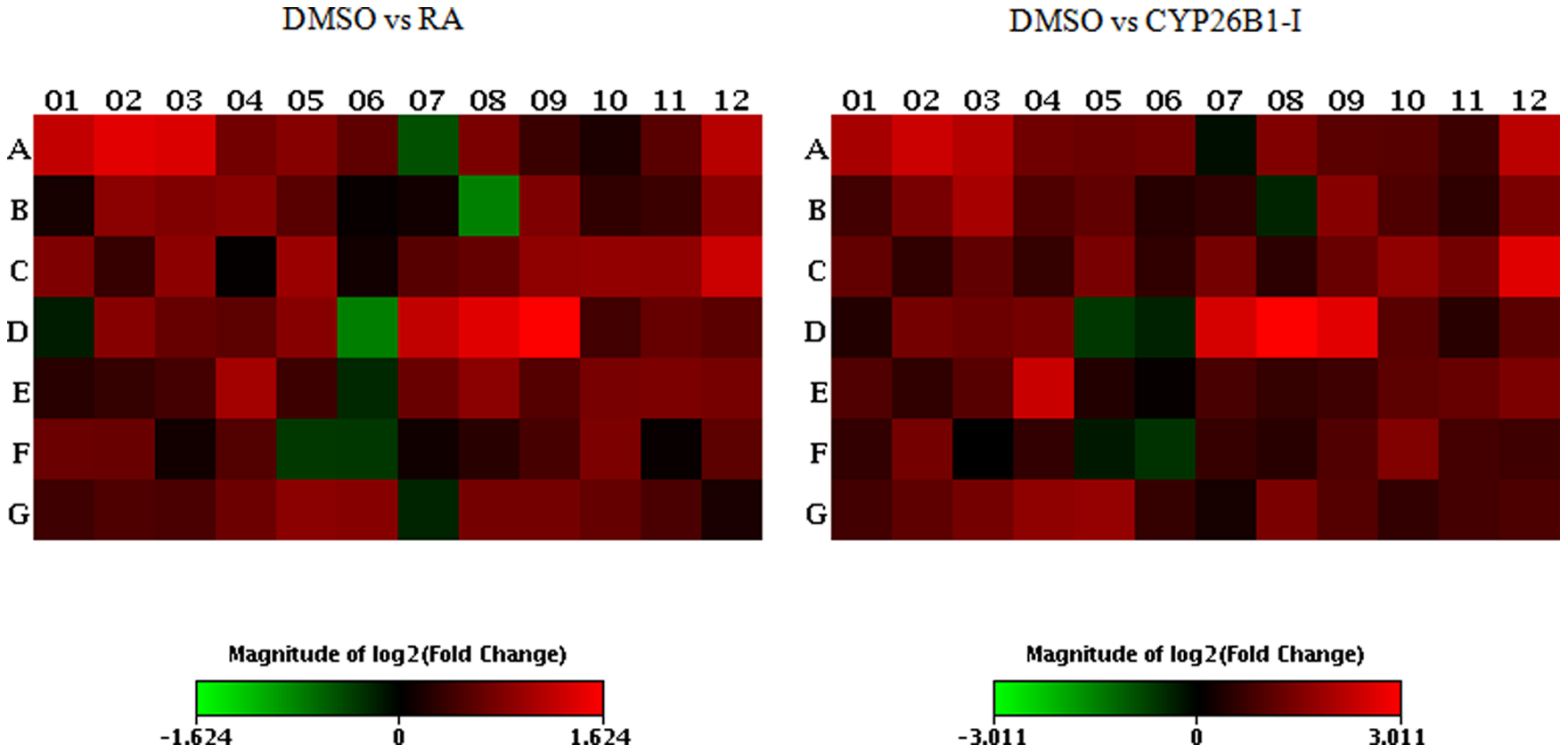
Heat Map of canine testis miRNA expression (Log transformed fold change) showing the pattern of RA or CYP26B1 inhibitor treated groups, related to the control (DMSO) group. Red indicates enhanced miRNA expression. Green indicates reduced miRNA expression (Refer to the Table S1 for well numbers and respective miRNA IDs).

**Figure 3:**
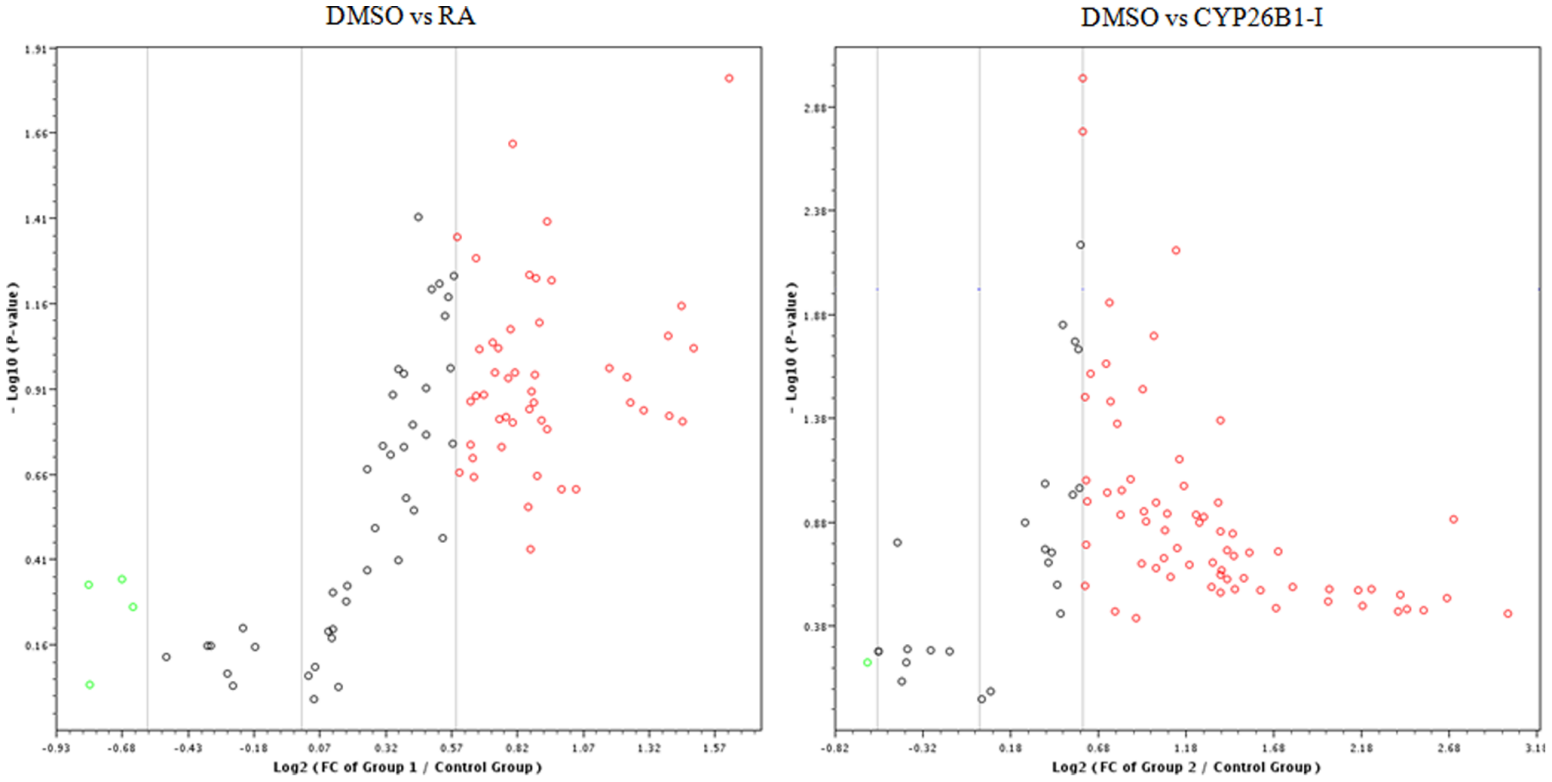
Volcano plots showing log 2 transformed related fold regulation of canine miRNA (RA or CYP26B1 inhibitor treatment related to control (DMSO). Red dots indicate up-regulated genes and green dots down-regulated genes. Outputs are obtained for the p-value of 0.01

**Figure 4:**
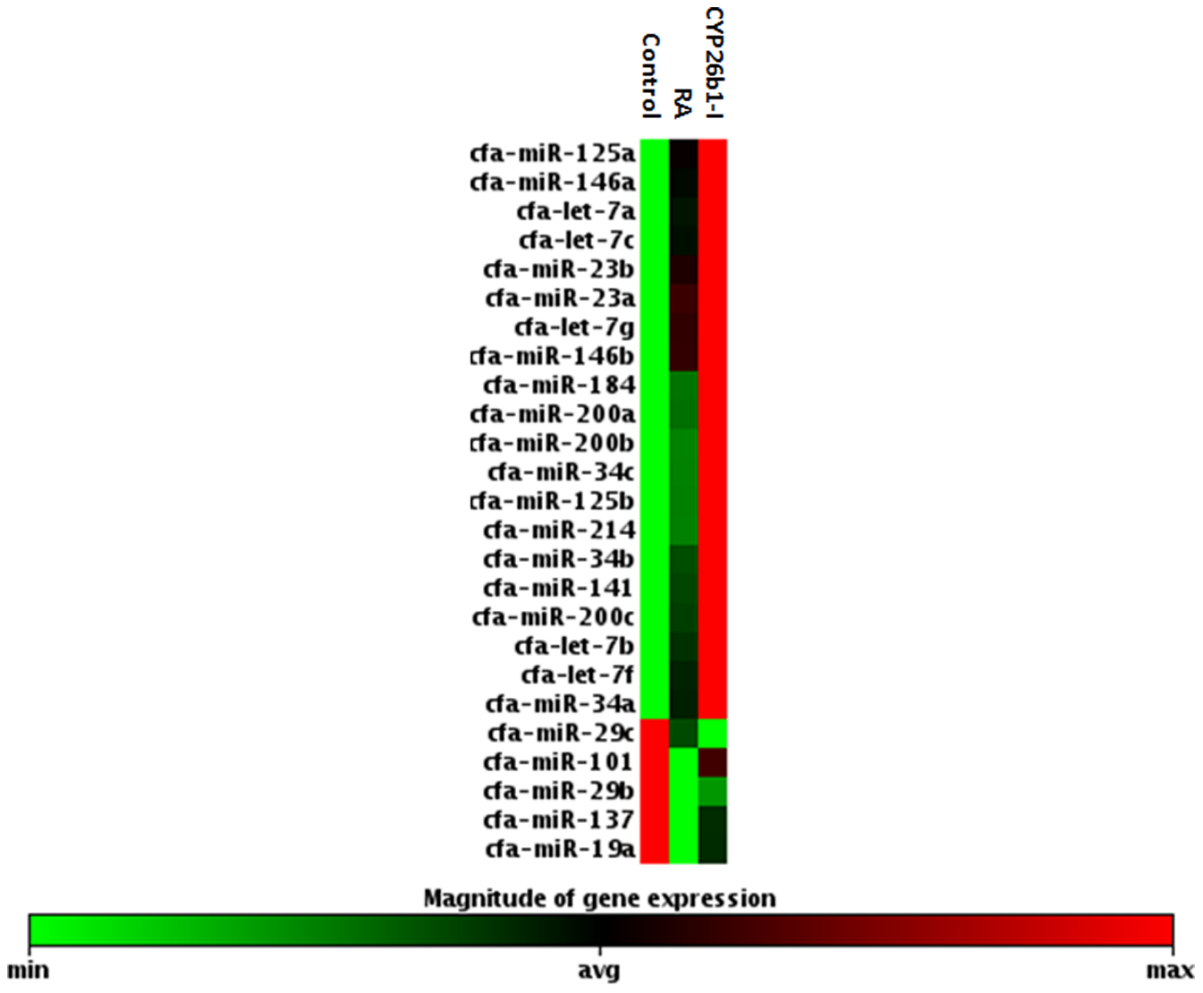
Clustergram of modulated miRNAs. Significantly up-regulated and down-regulated miRNAs were selected. DMSO (Control), RA and CYP26B1 treated groups are shown. Red indicates higher magnitude of miRNA expression whereas green indicates lower magnitude of miRNA expression.

**Figure 5:**
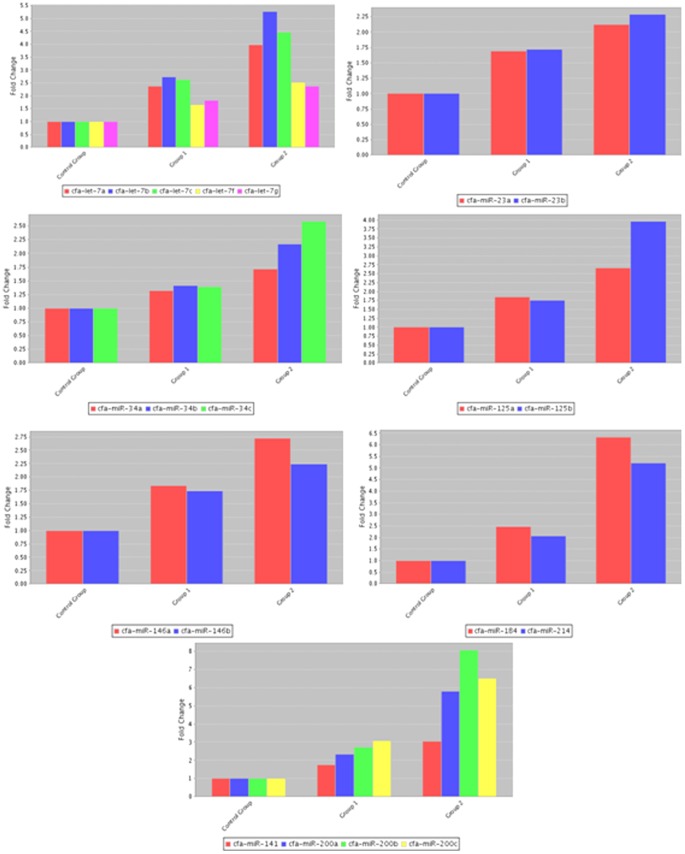
Up-regulated miRNAs (cfa-let-7, cfa-miR-200, cfa-miR-125, cfa-miR-34, cfa-miR-23, cfa-miR-146 clusters, cfa-miR-184 and cfa-miR-214) in adult canine testis treated with DMSO, RA or CYP26B1 inhibitor. Group 1 – RA treatment; Group 2 – CYP26B1 inhibitor treatment.

**Figure 6:**
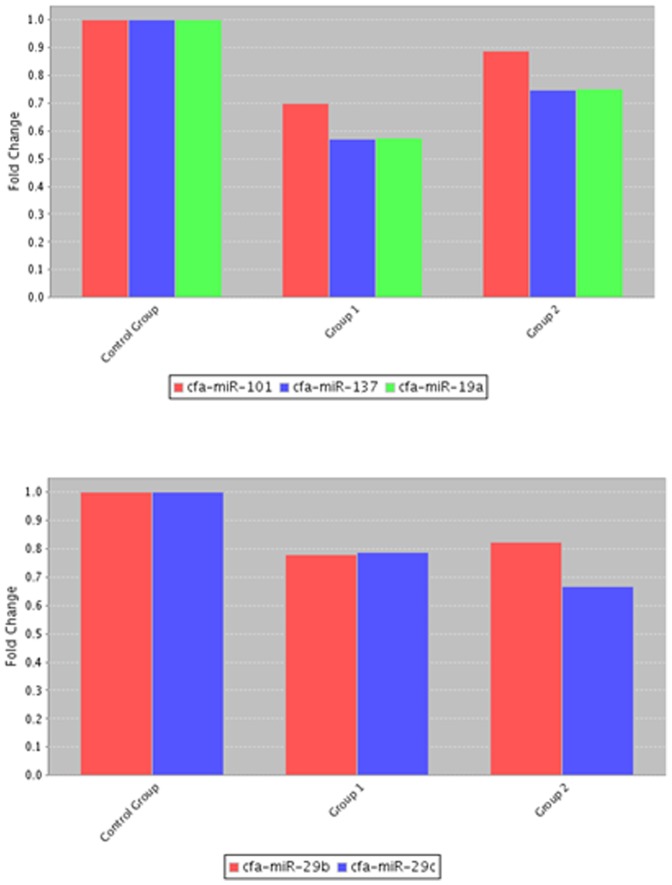
Down-regulated miRNAs (cfa-miR-29 cluster, cfa-miR-19a, cfa-miR-101 and cfa-miR-137) in adult canine testis treated with DMSO, RA (Group 1) or CYP26B1 inhibitor (Group 2).

MiRNA is a group of small non-encoding RNA molecules of 21–23 nucleotides in length, which controls gene expression post-transcriptionally either via the degradation of target mRNAs or the inhibition of protein translation [16]. Since the discovery of miRNA in 1993, 1872 precursors, 2578 mature human miRNAs have been added to the database (http://www.mirbase.org/). For Canis familiaris, 324 miRNA precursors and 291 mature miRNAs have been included in the database as well and chromosomes from which a number of miRNAs transcribed in canine species are now presented (Figure 1). To address our aim, we investigated 84 well-characterized canine mature miRNAs using a PCR-based canine specific miRNome array. The findings showed altered expression of miRNAs in response to intervention of RA signaling pathway in canine testis and suggest the regulatory role of miRNAs in the RA-mediated spermatogenesis. The RA and CYP26B1 inhibitor treatments modulated the miRNAs abundances compared to controls that had DMSO administration (Images of heatmap (Figure 2), Volcano plots (Figure 3), and clustergram (Figure 4) are presented). MiRNA families such as miR-200 (cfa-miR-200a, cfa-miR-200b and cfa-miR-200c), Mirlet-7 (cfa-let-7a, cfa-let-7b, cfa-let-7c, cfa-let-7g and cfa-let-7f), miR-125 (cfa-miR-125a and cfa-miR-125b), miR-146 (cfa-miR-146a and cfa-miR-146b), miR-34 (cfa-miR-34a, cfa-miR-34b and cfa-miR-34c), miR-23 (cfa-miR-23a and cfa-miR-23b), cfa-miR-184, cfa-miR-214 and cfa-miR-141 were significantly up-regulated with testicular RA intervention via administration of CYP26B1 inhibitor and all-trans-RA (Figure 5). Up-regulation of miRNA clusters was significantly higher with the treatment of CYP26B1 inhibitor, compared to the treatment of RA. This result is consistent with our previous study [6]. In our previous study, CYP26B1 inhibitor better modulated meiosis-associated and male germ cells specific genes, compared to RA administration. Species of miRNA which were significantly down-regulated were cfa-miR-19a, cfa-miR-29b, cfa-miR-29c, cfa-miR-101 and cfa-miR-137 (Figure 6). Interestingly, we noted that nucleotide sequences of these mature miRNAs are similar to the sequences of human mature miRNAs which is presented in Table 1.

**Table 1:** Dysregulated canine mature miRNA sequences and human orthologs.

CAUCUUACCGGACAGUGCUGGA---cfa-miR-200a (by similarity) CAUCUUACCGGACAGUGCUGGA---hsa-miR-200a-5p
CAUCUUACUGGGCAGCAUUGGA---cfa-miR-200b (by similarity) CAUCUUACUGGGCAGCAUUGGA---hsa-miR-200b-5p
UAAUACUGCCGGGUAAUGAUGGA---cfa-miR-200c (experimental) UAAUACUGCCGGGUAAUGAUGGA---hsa-miR-200c-3p
AACACUGUCUGGUAAAGAUGG---cfa-miR-141 (by similarity) UAACACUGUCUGGUAAAGAUGG---hsa-miR-141-3p
UGAGGUAGUAGGUUGUAUAGUU---cfa-let-7a (by similarity) UGAGGUAGUAGGUUGUAUAGUU---hsa-let-7a-5p
UGAGGUAGUAGGUUGUGUGGUU---cfa-let-7b (not experimental) UGAGGUAGUAGGUUGUGUGGUU-hsa-let-7b-5p
UGAGGUAGUAGGUUGUAUGGUU---cfa-let-7c (experimental) UGAGGUAGUAGGUUGUAUGGUU---hsa-let-7c-5p
UGAGGUAGUAGAUUGUAUAGUU---cfa-let-7f (experimental) UGAGGUAGUAGAUUGUAUAGUU---hsa-let-7f-5p
UGAGGUAGUAGUUUGUACAGUU---cfa-let-7g (experimental) UGAGGUAGUAGUUUGUACAGUU---hsa-let7g-5p
UCCCUGAGACCCUUUAACCUGU---cfa-miR-125a (experimental) UCCCUGAGACCCUUUAACCUGUGA---hsa-miR-125a-5p
UCCCUGAGACCCUAACUUGUGA---cfa-miR-125b (experimental) UCCCUGAGACCCUAACUUGUGA---hsa-miR-125b-5p
UGAGAACUGAAUUCCAUGGGUU---cfa-miR-146a (experimental) UGAGAACUGAAUUCCAUGGGUU---hsa-miR-146a-5p
UGAGAACUGAAUUCCAUAGGCU---cfa-miR-146b (experimental) UGAGAACUGAAUUCCAUAGGCU---hsa-miR-146b-5p
AUCACAUUGCCAGGGAUUU---cfa-miR-23a (experimental) AUCACAUUGCCAGGGAUUUCC---hsa-miR-23a-3p
AUCACAUUGCCAGGGAUUA---cfa-miR-23b (experimental) AUCACAUUGCCAGGGAUUACC---hsa-miR-23b-3p
UGGCAGUGUCUUAGCUGGUUGU---cfa-miR-34a (experimental) UGGCAGUGUCUUAGCUGGUUGU---hsa-miR-34a-5p
AGGCAGUGUAAUUAGCUGAUUG---cfa-miR-34b (by similarity) UAGGCAGUGUCAUUAGCUGAUUG---hsa-miR-34b-5p
AGGCAGUGUAGUUAGCUGAUUGC---cfa-miR-34c (experimental) AGGCAGUGUAGUUAGCUGAUUGC---hsa-miR-34c-5p
UGUGCAAAUCUAUGCAAAACUGA---cfa-miR-19a (experimental) UGUGCAAAUCUAUGCAAAACUGA---hsa-miR-19a-3p
UAGCACCAUUUGAAAUCAGUGUU---cfa-miR-29b (experimental) UAGCACCAUUUGAAAUCAGUGUU---hsa-miR-29b-3p
UAGCACCAUUUGAAAUCGGUUA---cfa-miR-29c (experimental) UAGCACCAUUUGAAAUCGGUUA---hsa-miR-29c-3p
UACAGUACUGUGAUAACUGA---cfa-miR-101 (experimental) UACAGUACUGUGAUAACUGAA---hsa-miR-101-3p
UUAUUGCUUAAGAAUACGCGU---cfa-miR-137 (experimental) UUAUUGCUUAAGAAUACGCGUAG---hsa-miR-137-3p
UGGACGGAGAACUGAUAAGGGU---cfa-miR-184 (by similarity) UGGACGGAGAACUGAUAAGGGU---hsa-miR-184
ACAGCAGGCACAGACAGGCAGU---cfa-miR-214 (by similarity) ACAGCAGGCACAGACAGGCAGU---hsa-miR-214-3p

Evidence of the addition of canine sequences to the database by experimental approach or by similarity for canine sequences is included in the table.

In an analysis of miRNA expression during primordial male germ cell development from embryonic day, E9.5 to E13.5 in mice, miR-200a, miR-200b and miR-141 expressions steadily decrease and reach the lowest level at the E13.5 [17]. The embryonic days associate with germ cells commitment to spermatogenesis at E12.5 and mitotic arrest at E13.5 [18]. Retinoic acid signaling is critical for the male germ cell commitment and spermatogenesis. RA metabolizing enzyme, CYP26B1 expression, regulating the cellular RA level is significantly seen in embryonic testis, but not in embryonic ovary. In the present study both exogenous administration of RA and CYP26B1 inhibitor significantly up-regulated the expressions of cfa-miR-200a, cfa-miR-200b, cfa-miR-200c and cfa-miR-141 and reveals the involvement of these miRNAs with RA-dependent spermatogenesis. Cfa-miR-200a, cfa-miR-200b, cfa-miR-200c and cfa-miR-141 are clustered together in the same family. Cfa-miR-200a and cfa-miR-200b are transcribed from chromosome 5 and cfa-miR-200c and cfa-miR-141 are transcribed from chromosome 27. In mammals, miR-34 miRNA family members were discovered computationally and then verified experimentally. In dogs, cfa-miR-34a, cfa-miR-34b and cfa-miR-34c are transcribed from regions of chromosome 5. In murine testis, miR-34a, miR-34b and miR-34c are expressed from post-natal day 11 (P11) and the expression is increased steadily to its highest enrichment at P60. The P11 is the critical time for spermatogenesis and testicular development in mice, during which time the expression of RA synthesizing enzyme begins to increase and RA degrading enzyme remains low. It is also the time for mitotic resumption, up-regulation of meiotic markers and meiotic entry. RA is the critical signal for the induction of meiosis at post-natal day 10 in mice. These previous findings together support the association of miR-34 clusters, RA signaling and murine spermatogenesis. In the present investigation, cfa-miR-34 clusters have been up-regulated significantly by exogenous RA and CYP26B1 inhibitor in canine testis. It has also been observed that enhancement of miR-34 cluster is higher with the CYP26B1 inhibitor treatment than direct RA administration. The results of the current study support the role of cfa-miR-34 clusters with RA-induced spermatogenesis in dogs. Additionally, a number of previous studies demonstrated that several miRNA species including miR-34 cluster regulate murine spermatogenesis. Mir-21, mir-34c and mir-221/222 control self-renewal of undifferentiated spermatogonia [19], Mirc1, Mirc3 and Mirlet7 regulate spermatogonial differentiation [9], [20], mir-15a and mir-184 mediate differentiation of spermatocytes [18], [19], miR-18, miR-34b, miR-34c, miR-184, miR-383, miR-449 and miR-469 mediate meiotic division of spermatocytes to spermatids [21], [23]–[25] and miR-469, miR-34c regulate differentiation of spermatid to form spermatozoa[24], [25].

In this study, canine let-7 family was significantly up-regulated by exogenous RA and CYP26B1 inhibitor in canine testis. A previous study [9] reported that mirlet-7 family of miRNAs is expressed in murine spermatogonia and spermatocytes and exogenous RA enhanced the expression of this family in spermatogonial cell culture. Testicular RA increase might regulate let-7 family through the pluripotency factor Lin28 protein since the same study noted the suppression of Lin28 by exogenous RA. Prominently, RNA binding protein, Lin28 has been shown to bind to the terminal loops of the precursors of miRNAs of the let-7 family, thereby blocking their processing into mature miRNAs. A recent study [26], observed that Lin 28 has negative feedback regulation on let-7 family of miRNAs in human gestational tissues. Let-7 family was one among high abundant miRNAs seen in human testis [27] and its target genes were integrated to canonical signaling pathways of spermatogenesis.

MiR-184 has been shown to be highly expressed in murine testis. An earlier study [22], demonstrated that miR-184 level was steadily increased during post-natal testicular development in mice. The same study also suggested that miR-184 down-regulates NCOR2 (nuclear receptor co-repressor 2) by targeting its 3 prime untranslated region and preventing NCOR2 protein translation. NCOR2 repression is necessary for RA signaling cascade and RA ligand is critical for regulation of spermatogenesis. Interestingly, in the present study cfa-miR-184 has been significantly up-regulated in response to direct and indirect increase of RA level in canine testis. Therefore, it is suggested that cfa-miR-184 is an RA-dependent regulator for canine spermatogenesis. Earlier studies [28], [29] reported that miR-214 in mice and mmu-miR-214 in pigs are significantly over-expressed in sexually immature testis compared to mature testis. However, our study has identified cfa-miR-214 as an RA pathway associated miRNA in canine spermatogenesis. In support of our findings, there is another study showed up-regulation of miR-214 in RA-induced differentiated ES cells [30].

A previous investigation [31] on miRNAs analyses with fetal gonad development identified that miR-101 is expressed in ovine fetal testis at gestational day 42 and not expressed at gestational day 75. Another study [32] identified the increase of miR-101 in differentiating chicken ovary and suggested that miR-101 can repress TGF-β signaling in that tissue. In our study, cfa-miR-101 has been down-regulated significantly by direct RA administration, but not by CYP26B1 inhibitor. The cfa-miR-101 may not have seed regions on target genes needed for spermatogenesis or RA-induced spermatogenesis. In this study, cfa-miR-19a has been considerably down-regulated following RA treatment and CYP26B1 inhibitor treatment. A previous study [17] predicted that miR-19a targets the tumor suppressor Phosphatase and tensin homolog (PTEN), and PTEN has a negative effect on proliferation of primordial germ cells. Considering our result and this previous report, it can be suggested that cfa-miR-19a is required for proliferation of primordial germ cells during the early embryonic development.

**Figure 7:**
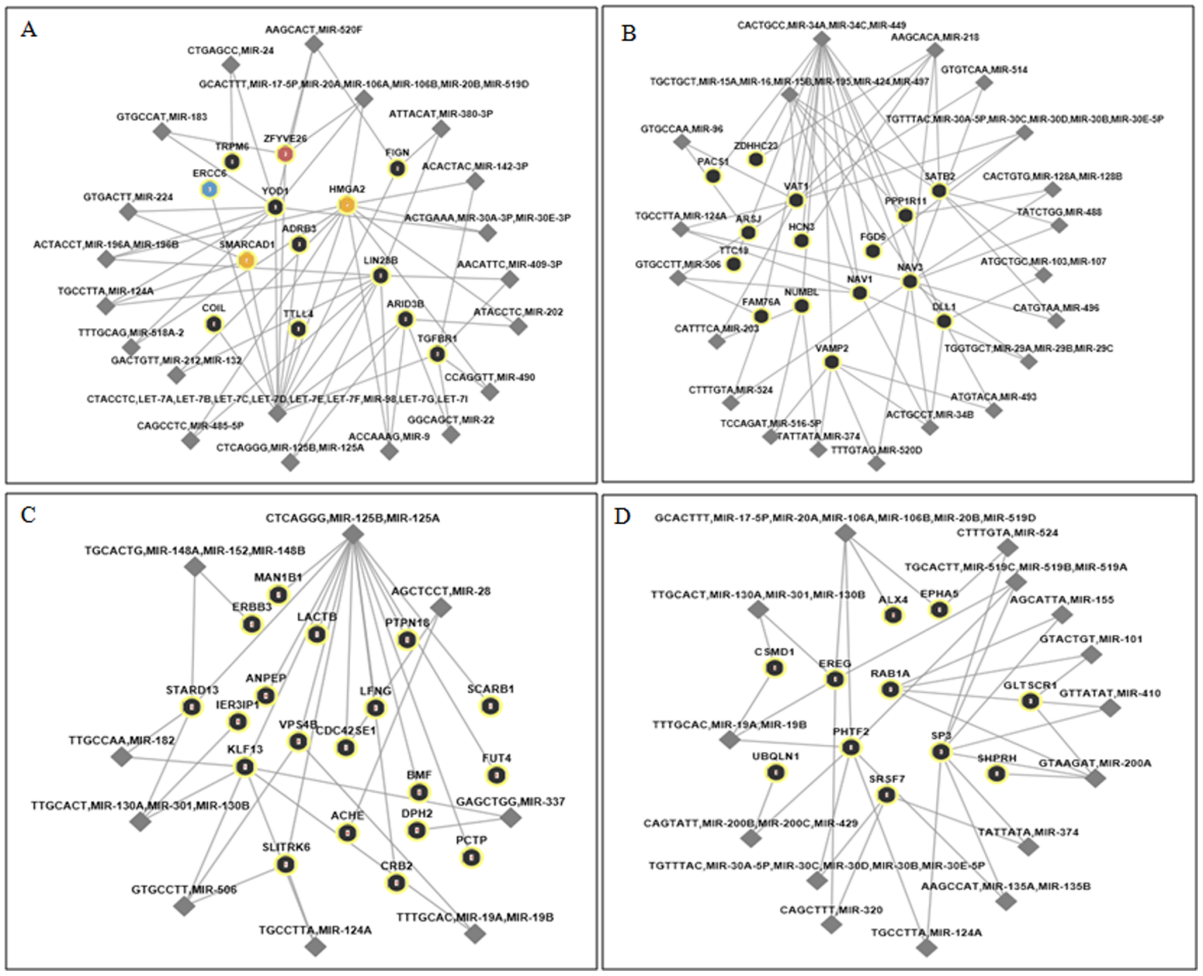
Integration network of miRNAs and genes. Let-7, miR-200, miR-34 and miR-125 clusters are chosen to create networks. Genes with the high context score are selected. Circles indicate genes and gene IDs are presented. Diamond shapes designate miRNAs and miRNAs' IDs are given. Other related miRNAs are also dragged into the network.

The integrative analyses of miRNAs and related genes using target prediction tools and network association tools have revealed that several miRNAs have seed regions on one gene and one miRNA has target sites on many genes (Figure 7). Among dysregulated miRNAs in this study, an association network was created for let-7, miR-200, miR-34 and miR-125 families. Of the interaction analyses, let-7 cluster interacted with HMGA2 (high mobility group AT-hook 2), LIN28B (lin-28 homolog B), ARID3B (AT rich interactive domain 3B), TTLL4 (tubulin tyrosine ligase-like family, member 4), ZFYVE26 (zinc finger, FYVE domain containing 26), ADRB3 (adrenergic, beta-3-, receptor), FIGN (fidgetin), SMARCAD1 (SWI/SNF-related, matrix-associated actin-dependent regulator of chromatin, subfamily a, containing DEAD/H box 1), TGFBR1 (transforming growth factor, beta receptor 1), YOD1 (YOD1 OTU deubiquinating enzyme 1 homolog), ERCC6 (excision repair cross-complementing rodent repair deficiency, complementation group 6), TRPM6 (transient receptor potential cation channel, subfamily M, member 6) and COIL (coilin) target genes. Genes with high total context score were included to draw the network. It was also noted that other miRNAs have integration into the same target genes. MiR-200 cluster had integration with the genes, such as SP3 (Sp3 transcription factor), EPHA5 (EPH receptor A5), EREG (epiregulin), ALX4 (ALX homeobox 4), GLTSCR1 (glioma tumor suppressor candidate region gene 1), SHPRH (SNF2 histone linker PHD RING helicase), CSMD1 (CUB and Sushi multiple domains 1), RAB1A (RAB1A, member RAS oncogene family), SRSF7 (serine/arginine-rich splicing factor 7), UBQLN1 (ubiquilin 1) and PHTF2 (putative homeodomain transcription factor 2). Target genes and their biological functions for miR-34 and miR-125 clusters are given in Table S2.

These inferences were based on human orthologous genes interpreted for canine miRNAs. Canine studies are needed for complete miRNA profiling and classification of miRNAs. Comprehensive investigations will aid to elucidate the integration of miRNA, mRNA and biological processes for canine spermatogenesis.

However, in other mammalian species, many of these genes are associated with spermatogenesis or shown to be expressed in testis. Some of these transcripts are highly conserved in several organs and perform global functions such as chromatin processing, ion transport and cell division and cycle. HMGA2 gene plays a crucial role in male fertility and its expression is related to murine spermatogenesis, especially seen during the transformation of spermatocyte to spermatid [33]. Gene and protein expressions of HMGA2 are observed in murine testicular cell lines [34]. LIN28 and LIN28B are differentially expressed in mouse testis during the post-natal development [35]. ARID3B is expressed in embryonic stem cells and in testis and is involved in cell proliferation and cell cycle progression [36]. Mammalian ZFY genes are located in the Y chromosome and several isoforms are expressed mainly in the testis [37]. Zinc finger protein negatively regulates the androgen receptor in the mouse testis via transcriptional regulation and the loss of the protein disrupts AR signaling [38]. Fidgetin regulates somatic cell mitosis and cell cycle [39]. SMARCAD1 is required for global deacetylation of histones, methylation of lysines, establishment of heterochromatin and chromosome segregation [40]. TGFBR1 has a ubiquitous expression pattern and is expressed in testes [41] and testicular expression of TGFBR1 is decreased in male lambs that were prenatally exposed to testosterone propionate compared to control male lambs [42]. YOD1 is a highly conserved deubiquitinating enzyme and maintains protein homeostasis in the endoplasmic reticulum. TRPM6 is generally required for cation transport and might play roles in calcium and magnesium ion transport in spermatozoa. Coilin, the cajal body marker protein is needed for viability, fertility and fecundity in mice [43]. SP3 transcription factor is abruptly decreased during mouse spermatogenesis [44]. SP3 is one of the Kruppel family proteins and Kruppel family proteins mediate a tight junction protein, CXADR-Like Membrane Protein (CLMP, adipocyte adhesion molecule) in murine Sertoli cells [45]. UBQLN1 interacts with Spermatid Maturation (SPEM) 1 and participates in spermiogenesis by regulating protein ubiquitination in mouse testis [46]. PHTF2 is expressed in mammalian testis [47]. Similarly, target genes and transcription factors of other modulated miRNAs following CYP26B1 and RA treatments in this study either correlate explicitly to spermatogenesis or connect to general functions such as chromatin conformation, epigenetic regulation and cell division.

## Conclusions

Thousands of miRNAs have been discovered and more are expected to be discovered in the near future. The discovery of miRNAs certainly has its impact on the field of reproduction. Essential roles in male reproduction have already been related via Drosha and Dicer dependent pathways. Global loss of miRNAs had detrimental effects on male fertility. Our study offers substantial information on miRNAs integrated with RA-dependent spermatogenesis in dogs. Yet, more in depth analyses of individual miRNA at the molecular level are necessary to identify its key roles in spermatogenesis. Identifying target genes and thus understanding the regulation of spermatogenesis by the miRNA clusters are now feasible with the generation of individual miRNA knock-outs and availability of miRNA mimics, enhancers and silencers.

## Supporting Information

Table S1 **miScript miRNA PCR Array Dog miFinder (MIFD-001Z), well number and respective miRNA IDs.**
(DOCX)

Table S2 **Associated genes and their biological function for miR34 and miR125 clusters (with references).**
(DOCX)
