# Supplementary material for: Dysregulated microRNA Clusters in Response to Retinoic Acid and CYP26B1 Inhibitor Induced Testicular Function in Dogs
Source: PLoS One. 2014 Jun 9;9(6):e99433. doi: 10.1371/journal.pone.0099433 (PMC4049822; doi:10.1371/journal.pone.0099433)
Supplement: Table S1 — miScript miRNA PCR Array Dog miFinder (MIFD-001Z), well number and respective miRNA IDs. (DOCX) [file pone.0099433.s001.docx]

Table S1

| Array Layout | | | | | | | | | | | |
| --- | --- | --- | --- | --- | --- | --- | --- | --- | --- | --- | --- |
| cfa-let-7a A01 | cfa-let-7b A02 | cfa-let-7c A03 | cfa-let-7f A04 | cfa-let-7g A05 | cfa-miR-1 A06 | cfa-miR-101 A07 | cfa-miR-103 A08 | cfa-miR-106a A09 | cfa-miR-106b A10 | cfa-miR-10b A11 | cfa-miR-122 A12 |
| cfa-miR-124 B01 | cfa-miR-125a B02 | cfa-miR-125b B03 | cfa-miR-126 B04 | cfa-miR-130a B05 | cfa-miR-133a B06 | cfa-miR-133b B07 | cfa-miR-137 B08 | cfa-miR-141 B09 | cfa-miR-143 B10 | cfa-miR-145 B11 | cfa-miR-146a B12 |
| cfa-miR-146b C01 | cfa-miR-148a C02 | cfa-miR-150 C03 | cfa-miR-15a C04 | cfa-miR-15b C05 | cfa-miR-16 C06 | cfa-miR-17 C07 | cfa-miR-181a C08 | cfa-miR-181b C09 | cfa-miR-182 C10 | cfa-miR-183 C11 | cfa-miR-184 C12 |
| cfa-miR-18a D01 | cfa-miR-191 D02 | cfa-miR-192 D03 | cfa-miR-195 D04 | cfa-miR-196a D05 | cfa-miR-19a D06 | cfa-miR-200a D07 | cfa-miR-200b D08 | cfa-miR-200c D09 | cfa-miR-203 D10 | cfa-miR-204 D11 | cfa-miR-205 D12 |
| cfa-miR-20a E01 | cfa-miR-21 E02 | cfa-miR-210 E03 | cfa-miR-214 E04 | cfa-miR-218 E05 | cfa-miR-22 E06 | cfa-miR-222 E07 | cfa-miR-223 E08 | cfa-miR-224 E09 | cfa-miR-23a E10 | cfa-miR-23b E11 | cfa-miR-24 E12 |
| cfa-miR-25 F01 | cfa-miR-26a F02 | cfa-miR-27a F03 | cfa-miR-27b F04 | cfa-miR-29b F05 | cfa-miR-29c F06 | cfa-miR-30b F07 | cfa-miR-30c F08 | cfa-miR-30d F09 | cfa-miR-31 F10 | cfa-miR-335 F11 | cfa-miR-342 F12 |
| cfa-miR-34a G01 | cfa-miR-34b G02 | cfa-miR-34c G03 | cfa-miR-375 G04 | cfa-miR-378 G05 | cfa-miR-451 G06 | cfa-miR-499 G07 | cfa-miR-7 G08 | cfa-miR-9 G09 | cfa-miR-92a G10 | cfa-miR-93 G11 | cfa-miR-96 G12 |
| cel-miR-39-3p H01 | cel-miR-39-3p H02 | SNORD61 H03 | SNORD68 H04 | SNORD72 H05 | SNORD95 H06 | SNORD96A H07 | RNU6-2 H08 | miRTC H09 | miRTC H10 | PPC H11 | PPC H12 |
