# Supplementary material for: Dysregulated microRNA Clusters in Response to Retinoic Acid and CYP26B1 Inhibitor Induced Testicular Function in Dogs
Source: PLoS One. 2014 Jun 9;9(6):e99433. doi: 10.1371/journal.pone.0099433 (PMC4049822; doi:10.1371/journal.pone.0099433)
Supplement: Table S2 — Associated genes and their biological function for miR34 and miR125 clusters (with references). (DOCX) [file pone.0099433.s002.docx]

Table S2

| miRNA family | Gene | Biological function | Species | Ref |
| --- | --- | --- | --- | --- |
| miR-34 | Zinc finger, DHHC-type containing 23 (ZDHHC23) | Regulation of ion channels | Human, mouse, rat, cow, dog, chicken, rhesus monkey, camel and alpaca | 1 |
|  | Phosphofurin acidic cluster sorting protein 1 (PACS1) | Apoptosis | Human, mouse, rat  cow, dog, chicken, rhesus monkey, camel and alpaca | 2,3 |
|  | Vesicle amine transport 1 (VAT1) | Phosphorylation | Human, mouse, rat,  cow, pig, dog, chicken, rhesus monkey, camel and alpaca | 4 |
|  | Arylsulfatase family, member J (ARSJ) | Calcium ion binding, metabolism; hydrolase activity | Human, mouse, rat,  cow, goat, dog, chicken, rhesus monkey, camel and alpaca | 5 |
|  | Tetratricopeptide repeat domain 15 (TTC15) | Transcription, cell cycle, protein translocation, protein degradation | Human, mouse, rat,  cow, dog, Chicken, rhesus monkey, ferret and guinea pig | 6 |
|  | Hyperpolarization activated cyclic nucleotide-gated potassium channel 3 (HCN3) | Regulation of the biophysical properties of the ion channel | Human, mouse, rat,  cow, goat, dog, chicken, rhesus monkey, camel and alpaca | 7 |
|  | Numb homolog (Drosophila)-like (NUMBL) | Spermatogenesis | Human, Rat, Mouse | 8 |
|  | Vesicle-associated membrane protein 2 (VAMP2) | Cell adhesion, migration and survival | Human, mouse, rat,  cow, dog, chicken, rhesus monkey, zebra fish and alpaca | 9 |
|  | Neuron navigator (NAV) 1 and 3 | Cytoskeleton | Human, rat, mouse, cow, dog, Rhesus monkey, chicken and zebra fish | 10 |
|  | F_420_-dependent glucose-6-phosphate dehydrogenase (FGD) | Cytoskeleton, cell morphology and motility | Human, rat, mouse, chicken and zebra fish | 11 |
|  | Protein phosphatase 1 regulatory subunit 11  (PPP1R11) | Germ cell apoptosis | Human, rat, mouse, cow, dog, pig, rhesus monkey, alpaca and camel | 12 |
|  | Special AT-rich sequence binding protein 2 (SATB2) | Cytoskeleton, proliferation of osteoblast like cell | Human, rat, mouse, cow, horse, sheep dog, chicken, rhesus monkey, alpaca and camel | 13 |
|  | Delta-like 1 (DLL1) | Notch signaling, angiogensis, homeostasis of stem cells | Human, rat, mouse, cow, dog, chicken, rhesus monkey, alpaca and camel | 14,15 |
| miR-125 | Mannosidase, alpha, class 1B, member 1 (MAN1B1) | Ubiquitination, glycosylation, hydrolysis | Human, rat, mouse, cow, dog, chicken, and rhesus monkey | 16 |
|  | V-Erb-B2 Avian Erythroblastic Leukemia Viral Oncogene Homolog (ERBB3) | Cell signaling, spermatogonial DNA synthesis, cell cycle | Human, rat, mouse, cow, horse, sheep dog, chicken, rabbit, rhesus monkey and panda | 17-19 |
|  | Lactamase, beta (LACTB) | Mitochondrial function, | Human, rat, mouse, cow, horse, sheep dog, chicken, rhesus monkey, alpaca and camel | 20 |
|  | StAR-related lipid transfer (START) domain containing 13 (STARD13) | Development, apoptosis, cell cycle arrest | Human, rat, mouse, cow, dog, chicken, and rhesus monkey | 21,22 |
|  | Immediate early response 3 interacting protein 1(IER3IP1) | Morphogenesis, cell growth | Human, mouse, cow, dog, zebra fish, alpacaand camel | 23 |
|  | Alanyl (membrane) aminopeptidase (ANPEP) | Angiogenesis, apoptosis | Human, rat, mouse, cow, pig, cat, dog, chicken, rhesus monkey, alpaca and camel | 24 |
|  | Kruppel-like factor 13 (KLF13) | Cell proliferation, differentiation, cell cycle progression, and apoptosis | Human, rat, mouse, cow, pig, dog, chicken, zebra fish and rhesus monkey | 25-27 |
|  | SLIT and NTRK-like family, member 6 (SLITRK6) | Development, biological process | Human, rat, mouse, cow, dog, pig, sheep, horse, chicken, Rhesus monkey, alpaca and camel | 28 |
|  | Acetylcholinesterase (ACHE) | Glycerophospholipid metabolism, apoptosis, cellular stress | Human, rat, mouse, cow, dog, cat, chicken, zebra fish and rhesus monkey | 29,30 |
|  | Vacuolar Protein Sorting 4 Homolog B (VPS4B) | Endocytosis, intracellular protein trafficking | Human, rat, mouse, cow, dog, chicken, rhesus monkey, camel and alpaca | 31,32 |
|  | CDC42 small effector 1 (CDC42SE1) | Regulation of cytoskeleton or in kinase signaling | Human, mouse, cow, dog, pig, chicken, zebra fish, rhesus monkey, camel and alpaca | 33 |
|  | LFNG O-fucosylpeptide 3-beta-N-acetylglucosaminyltransferase (LFNG) | Testis development | Human, mouse, cow, dog, goat chicken, zebra fish, camel and alpaca | 34 |
|  | Crumbs family member 2 (CRB2) | Histone methylation, DNA repair | Human, mouse, rat, cow, dog, chicken, zebra fish, camel and alpaca | 35 |
|  | DPH2 homolog (DPH2) | Cell proliferation, embryonic development | Human, mouse, rat, cow, dog, chicken, zebra fish, ferret and golden hamster | 36 |
|  | Bcl2 modifying factor (BMF) | Apoptosis | Human, mouse, rat, cow, dog, chicken, rhesus monkey, zebra finch, camel and alpaca | 37,38 |
|  | Phosphatidylcholine transfer protein (PCTP) | Postnatal development, sperm energy metabolism | Human, mouse, rat, cow, dog, chicken, ferret, rhesus monkey, zebra fish, camel and alpaca | 39 |
|  | Fucosyltransferase 4 (FUT4) | Gamete maturation, embryogenesis | Human, mouse, rat, cow, dog, chicken, camel and alpaca | 40,41 |
|  | Scavenger receptor class B, member 1 (SCARB1) | Embryonic development | Human, mouse, rat, cow, dog, pig, rhesus monkey, chicken, horse, camel and alpaca | 42, 43 |
|  | Protein tyrosine phosphatase, non-receptor type 18 (PTPN18) | Phosphorylation, cell function, cellular signaling | Human, mouse, rat, cow, dog, rhesus monkey, chicken, camel and alpaca | 44,45 |

1. Tian L, McClafferty H, Knaus HG, Ruth P, Shipston MJ (2012) Distinct acyl protein transferases and thioesterases control surface expression of calcium-activated potassium channels. J Biol Chem 287: 14718-14725.
2. Simmen T, Aslan JE, Blagoveshchenskaya AD, Thomas L, Wan L, et al (2005). PACS-2 controls endoplasmic reticulum-mitochondria communication and Bid-mediated apoptosis. EMBO J 24: 717-729.
3. Köttgen M, Benzing T, Simmen T, Tauber R, Buchholz B, et al (2005). Trafficking of TRPP2 by PACS proteins represents a novel mechanism of ion channel regulation. EMBO J 24: 705-716.
4. Faugaret D, Chouinard FC, Harbour D, El azreq MA, Bourgoin SG. An essential role for phospholipase D in the recruitment of vesicle amine transport protein-1 to membranes in human neutrophils. Biochem Pharmacol 81: 144-156.
5. Larsson O, Diebold D, Fan D, Peterson M, Nho RS, et al (2008) Fibrotic myofibroblasts manifest genome-wide derangements of translational control. PLoS One 3: e3220.
6. Allan RK, Ratajczak T (2011) Versatile TPR domains accommodate different modes of target protein recognition and function. Cell Stress Chaperones 16: 353-367.
7. Cao-Ehlker X, Zong X, Hammelmann V, Gruner C, Fenske S, et al (2013). Up-regulation of hyperpolarization-activated cyclic nucleotide-gated channel 3 (HCN3) by specific interaction with K+ channel tetramerization domain-containing protein 3 (KCTD3). J Biol Chem 288: 7580-7589.
8. Grisanti L, Corallini S, Fera S, Muciaccia B, Stefanini M, et al (2009). Inactivation of Numb and Numblike in spermatogonial stem cells by cell-permeant Cre recombinase. Differentiation 78: 131-136.
9. Hasan N, Hu C (2010) Vesicle-associated membrane protein 2 mediates trafficking of alpha5beta1 integrin to the plasma membrane. Exp Cell Res 316: 12-23.
10. van Haren J, Draegestein K, Keijzer N, Abrahams JP, Grosveld F, et al (2009) Mammalian Navigators are microtubule plus-end tracking proteins that can reorganize the cytoskeleton to induce neurite-like extensions. Cell Motil Cytoskeleton 66: 824-838.
11. Hayakawa M, Matsushima M, Hagiwara H, Oshima T, Fujino T, et al (2008) Novel insights into FGD3, a putative GEF for Cdc42, that undergoes SCF(FWD1/beta-TrCP)-mediated proteasomal degradation analogous to that of its homologue FGD1 but regulates cell morphology and motility differently from FGD1. Genes Cells 13: 329-342.
12. Cheng L, Pilder S, Nairn AC, Ramdas S, Vijayaraghavan S. PP1gamma2 and PPP1R11 are parts of a multimeric complex in developing testicular germ cells in which their steady state levels are reciprocally related. PLoS One 4: e4861.
13. Dobreva G, Chahrour M, Dautzenberg M, Chirivella L, Kanzler B, et al (2006) SATB2 is a multifunctional determinant of craniofacial patterning and osteoblast differentiation. Cell 125: 971-986.
14. Pellegrinet L, Rodilla V, Liu Z, Chen S, Koch U, et al (2011) Dll1- and dll4-mediated notch signaling are required for homeostasis of intestinal stem cells. Gastroenterology 140: 1230-1240.
15. Fernandez I, Ooi TP, Roy K. Generation of Functional, Antigen-Specific CD8+ Human T Cells from Cord Blood Stem Cells Using Exogenous Notch and Tetramer-TCR Signaling. Stem Cells 32: 93-104.
16. Rymen D, Peanne R, Millón MB, Race V, Sturiale L, et al (2013) MAN1B1 deficiency: an unexpected CDG-II. PLoS Genet 9: e1003989.
17. Abid SN, Richardson TE, Powell HM, Jaichander P, Chaudhary J, et al (2014) A-Single Spermatogonia Heterogeneity and Cell Cycles Synchronize with Rat Seminiferous Epithelium Stages VIII-IX. Biol Reprod 90: 32.
18. Wahab-Wahlgren A, Martinelle N, Holst M, Jahnukainen K, Parvinen M, et al (2003) EGF stimulates rat spermatogonial DNA synthesis in seminiferous tubule segments in vitro. Mol Cell Endocrinol 201: 39-46.
19. Kierszenbaum AL, Tres LL. Primordial germ cell-somatic cell partnership: a balancing cell signaling act. Mol Reprod Dev 60: 277-280.
20. Polianskyte Z, Peitsaro N, Dapkunas A, Liobikas J, Soliymani R, et al (2009) LACTB is a filament-forming protein localized in mitochondria. Proc Natl Acad Sci U S A 106: 18960-18965.
21. Petzold KM, Naumann H, Spagnoli FM. Rho signalling restriction by the RhoGAP Stard13 integrates growth and morphogenesis in the pancreas. Development 140: 126-135.
22. El-Sitt S, Khalil BD, Hanna S, El-Sabban M, Fakhreddine N, et al (2012) DLC2/StarD13 plays a role of a tumor suppressor in astrocytoma. Oncol Rep 28: 511-518.
23. Wang WJ, Zhang Y, Huang FX (2007) The expression of IER3IP1 gene in K562 cells treated by matrine and its effect on the cell growth. Zhonghua Xue Ye Xue Za Zhi 28: 823-827.
24. Sjöström H, Norén O, Olsen J (2000) Structure and function of aminopeptidase N. Adv Exp Med Biol 477: 25-34.
25. Chen XM, Johns DC, Geiman DE, Marban E, Dang DT, et al (2001) Krüppel-like factor 4 (gutenriched Krüppel-like factor) inhibits cell proliferation by blocking G(1)/S progression of the cell cycle. J Biol Chem 276: 30423-30428.
26. Kaczynski J, Cook T, Urrutia R (2003) Sp1-and Krüppel-like transcription factors. Genome Biol 4: 206.
27. Nemer M, Horb ME (2007) The KLF family of transcriptional regulators in cardiomyocyte proliferation and differentiation. Cell Cycle 6: 117-121.
28. Tekin M, Chioza BA, Matsumoto Y, Diaz-Horta O, Cross HE, et al (2013) SLITRK6 mutations cause myopia and deafness in humans and mice. J Clin Invest 123: 2094-2102.
29. Mor I, Sklan EH, Podoly E, Pick M, Kirschner M (2008) Acetylcholinesterase-R increases germ cell apoptosis but enhances sperm motility. Cell Mol Med 12: 479-495.
30. Mor I, Grisaru D, Titelbaum L, Evron T, Richler C, et al (2001) Modified testicular expression of stress-associated "readthrough" acetylcholinesterase predicts male infertility. FASEB J 15: 2039-2041.
31. Scheuring S, Röhricht RA, Schöning-Burkhardt B, Beyer A, Müller S, et al (2001) Mammalian cells express two VPS4 proteins both of which are involved in intracellular protein trafficking. Mol Biol 312: 469-480.
32. Piper RC, Luzio JP (2007) Ubiquitin-dependent sorting of integral membrane proteins for degradation in lysosomes. Curr Opin Cell Biol 19: 459-465.
33. Pirone DM, Fukuhara S, Gutkind JS, Burbelo PD (2000) SPECs, small binding proteins for Cdc42. J Biol Chem 275: 22650-22656.
34. Hahn KL, Beres B, Rowton MJ, Skinner MK, Chang Y et al (2009) A deficiency of lunatic fringe is associated with cystic dilation of the rete testis. Reproduction 137: 79-93.
35. Botuyan MV, Lee J, Ward IM, Kim JE, Thompson JR et al (2006) Structural basis for the methylation state-specific recognition of histone H4-K20 by 53BP1 and Crb2 in DNA repair. Cell 127: 1361-1373.
36. Chen CM, Behringer RR (2004) Ovca1 regulates cell proliferation, embryonic development, and tumorigenesis. Genes Dev 18: 320-332.
37. Grespi F, Soratroi C, Krumschnabel G, Sohm B, Ploner C et al (2010) BH3-only protein Bmf mediates apoptosis upon inhibition of CAP-dependent protein synthesis. Cell Death Differ 17: 1672-1683.
38. Madden DT, Davila-Kruger D, Melov S, Bredesen DE (2011) Human embryonic stem cells express elevated levels of multiple pro-apoptotic BCL-2 family members. PLoS One 6: e28530.
39. Yamanaka M, Koga M, Tanaka H, Nakamura Y, Ohta H et al (2000) Molecular cloning and characterization of phosphatidylcholine transfer protein-like protein gene expressed in murine haploid germ cells. Biol Reprod 62: 1694-1701.
40. Wang CM, Hu SG, Ru YF, Yao GX, Ma WB et al (2013) Different effects of androgen on the expression of Fut1, Fut2, Fut4 and Fut9 in male mouse reproductive tract. Int J Mol Sci 14: 23188-23202.
41. Cailleau-Thomas A, Coullin P, Candelier JJ, Balanzino L, Mennesson B et al (2000) FUT4 and FUT9 genes are expressed early in human embryogenesis. Glycobiology 10: 789-802.
42. Santander NG, Contreras-Duarte S, Awad MF, Lizama C, Passalacqua I et al (2013) Developmental abnormalities in mouse embryos lacking the HDL receptor SR-BI. Hum Mol Gene 22: 1086-1096.
43. Hatzopoulos AK, Rigotti A, Rosenberg RD, Krieger M (1998) Temporal and spatial pattern of expression of the HDL receptor SR-BI during murine embryogenesis. J Lipid Res 39: 495-508.
44. Zhang ZY (1998) Protein-tyrosine phosphatases: biological function, structural characteristics, and mechanism of catalysis. Crit Rev Biochem Mol Biol 33: 1-52.
45. Gandhi TK, Chandran S, Peri S, Saravana R, Amanchy R et al (2005) A bioinformatics analysis of protein tyrosine phosphatases in humans. DNA Res 12: 79-89.
